# Reproducibility of serum IgE, Ara h2 skin prick testing and fraction of exhaled nitric oxide for predicting clinical peanut allergy in children

**DOI:** 10.1186/s13223-016-0143-z

**Published:** 2016-08-05

**Authors:** Elizabeth Percival, Rani Bhatia, Kahn Preece, Patrick McElduff, Mark McEvoy, Adam Collison, Joerg Mattes

**Affiliations:** 1 Experimental & Translational Respiratory Medicine Group, Hunter Medical Research Institute, University of Newcastle, Lookout Road, New Lambton, Newcastle, NSW 2305 Australia; 2 Department of Paediatric Medicine, John Hunter Children’s Hospital, Newcastle, NSW Australia; 3 Department of Paediatric Allergy & Immunology, John Hunter Children’s Hospital, Newcastle, NSW Australia; 4 School of Medicine and Public Health, University of Newcastle, Newcastle, NSW Australia; 5 Department of Paediatric Immunology & Allergy, Starship Children’s Health, Auckland, New Zealand; 6 Department of Paediatric Respiratory & Sleep Medicine, John Hunter Children’s Hospital, Newcastle, NSW Australia

**Keywords:** Peanut, Allergy, Anaphylaxis, Predict, Reproducibility, Ara h2, Skin prick test, Fraction exhaled nitric oxide, Peanut sIgE, Ara h2 sIgE

## Abstract

**Background:**

Ara h2 sIgE serum levels improve the diagnostic accuracy for predicting peanut allergy, but the use of Ara h2 purified protein as a skin prick test (SPT), has not been substantially evaluated. The fraction of exhaled nitric oxide (FeNO) shows promise as a novel biomarker of peanut allergy. Reproducibility of these measures has not been determined. The aim was to assess the accuracy and reproducibility (over a time-period of at least 12 months) of SPT to Ara h2 in comparison with four predictors of clinical peanut allergy (Peanut SPT, Ara h2 specific Immunoglobulin E (sIgE), Peanut sIgE and FeNO).

**Methods:**

Twenty-seven children were recruited in a follow-up of a prospective cohort of fifty-six children at least 12 months after an open-labelled peanut food challenge. Their repeat assessment involved a questionnaire, SPT to peanut and Ara h2 purified protein, FeNO and sIgE to peanut and Ara h2 measurements.

**Results:**

Ara h2 SPT was no worse in accuracy when compared with peanut SPT, FeNO, Ara h2 sIgE and peanut sIgE (AUC 0.908 compared with 0.887, 0.889, 0.935 and 0.804 respectively) for predicting allergic reaction at previous food challenge. SPT for peanut and Ara h2 demonstrated limited reproducibility (ICC = 0.51 and 0.44); while FeNO demonstrated good reproducibility (ICC = 0.73) and sIgE for peanut and Ara h2 were highly reproducible (ICC = 0.81 and 0.85).

**Conclusions:**

In this population, Ara h2 SPT was no worse in accuracy when compared with current testing for the evaluation of clinical peanut allergy, but had—like peanut SPT—poor reproducibility. FeNO, peanut sIgE and Ara h2 sIgE were consistently reproducible despite an interval of at least 12 months between the repeated measurements.

**Electronic supplementary material:**

The online version of this article (doi:10.1186/s13223-016-0143-z) contains supplementary material, which is available to authorized users.

## Background

Peanut allergy can be a life threatening event and accounts for approximately two-thirds of all fatal food-induced anaphylaxis [1]. In a recent Australian population based study [2], the prevalence of peanut sensitisation [by skin prick testing (SPT)] in infants was 6.4 %, with a prevalence of clinical allergy (confirmed by oral food challenge) of 2.9 %. Furthermore, clinical peanut allergy resolves in up to 20 % of children [3] but the processes involved in resolution are not fully understood [4].

Current testing to confirm sensitisation to peanut includes SPT to peanut protein and specific immunoglobulin E (sIgE) antibodies to peanut [5]. The gold standard for diagnosing clinical allergy is a double-blind placebo-controlled oral food challenge [6]. However, there is the associated risk of severe allergic reaction (including anaphylaxis), financial cost to health care (to provide beds and supervision), and finally time involved for patients, their families and health care professionals. To alleviate this, in clinical practice it is routine to conduct open-labelled food challenges [2, 7] and to exclude children at extremely low or high risk for clinical allergy from food challenge employing (a set of) non-invasive biomarkers. For instance, the resulting size of SPT wheal to whole peanut antigen or levels of sIgE antibodies to peanut is thought to correlate with an increasing likelihood of reaction [8, 9], but not with increasing severity of the reaction at food challenge [6]. While these tests confirm an allergy based on a significant clinical history, they do not suggest how severe the reaction will be on subsequent exposures. Monitoring these values over time may assist with identifying patients who are likely to outgrow their allergies (decreasing size of SPT wheal). However, as SPT is an operator driven test this type of deduction may at times be erroneous, placing children at risk of allergic reaction at a food challenge.

Prospectively measured levels of serum sIgE against the peanut component Ara h2 have been investigated and when used in combination with peanut SPT, found to improve the diagnostic accuracy and reduce the need for oral peanut challenge [5, 7]. Two studies published in 2007 [10, 11] have investigated the use of Ara h2 purified protein as a SPT reagent, but there has been no further published data reporting on its use.

Fraction of exhaled nitric oxide (FeNO) is a non-invasive marker that has been shown to correlate with allergic airways inflammation and IgE sensitisation [12]. Additionally, there appears to be enhanced prediction of peanut allergy prior to food challenge when combining measurement of FeNO with current testing (peanut SPT and Ara h2 sIgE) [7].

The primary aim of this study was to assess the accuracy of purified Ara h2 protein SPT in comparison with four predictors of peanut allergy (peanut SPT, peanut sIgE, Ara h2 sIgE and FeNO). The secondary aim was to assess the reproducibility (over a time-period of at least 12 months) of Ara h2 SPT, in comparison with the same four predictors of peanut allergy, by following-up a population of children studied previously [7].

## Methods

### Study population

Twenty-seven children were able to be recruited in follow-up from a cohort of fifty-six children enrolled in an earlier prospective study [7] that had involved children scheduled for open-labelled peanut food challenge by their paediatric allergist at a tertiary referral paediatric allergy centre in Newcastle, Australia. Their food challenge in the initial study had been scheduled to (1) confirm a peanut allergy diagnosis, (2) assess for the possibility of acquired tolerance, (3) test for clinical reactivity in children who had not consumed peanut but were sensitised, or (4) had significant parental concern and anxiety [7]. Participants were excluded from the earlier study if their SPT to whole peanut extract was ≥10 mm [7].

The original cohort (of fifty-six children) included thirty-two participants with a history of IgE mediated reactions, including anaphylaxis, not within the previous 12 months. Three participants who underwent food challenge in the original study had equivocal challenge results and were excluded from the data analysis.

### Ethics and consent

The Hunter New England Health Human Research Ethics Committee approved both studies. Informed written consent was obtained from all parents or guardians prior to entry into the study, and from children as appropriate for their age. Participants from the earlier study were invited by telephone call from the Allergy Clinical Nurse Consultant to participate in this follow-up study.

### Original cohort assessment

Prior to the food challenge, the original cohort (of fifty-six children) was assessed in a pre-challenge clinic. This clinic included assessing personal atopy and family history of atopy by way of a modified version of a previously validated parental questionnaire [13, 14]. Allergic rhinitis was assessed using paediatric validated allergic rhinitis and its impact on asthma (ARIA) criteria [15], where classification is according to symptom duration (intermittent or persistent) and severity (mild or moderate/severe). Each was then scored 1—intermittent mild, 2—intermittent moderate/severe, 3—persistent mild or 4—persistent moderate/severe. Eczema was assessed based on any previous medical diagnosis, and current “active treatment” including any current management other than emollients. Visible eczema was scored using the validated SCORing Atopic Dermatitis (SCORAD) system [16].

Patients in the original cohort then underwent SPT which was performed on the volar surface of the patient’s forearm using standard whole peanut extract reagent, 1:10 w/v (Stallergenes, Antony, France). A positive result was ≥3 mm determined by averaging maximal perpendicular wheal diameters fifteen minutes after applying the lancet. Positive control was with histamine base, 6 mg/mL (Stallergenes, Antony, France) and with a wheal ≥3 mm indicating a valid test [17]. Negative control was glycerol saline. Later in the study, a small number were able to be skin prick tested with purified protein Ara h2 (100 μg/mL in glycerol saline solution). The Ara h2 protein was sourced commercially from Protein Labs, San Diego, California, where it was purified from peanut extract.

Serum was collected and analysed using ImmunoCAP 250 system (Phadia, AB, Uppsala, Sweden) for peanut sIgE and Ara h2-specific IgE.

FeNO was measured according to the American Thoracic Society and European Respiratory Society (ATS/ERS) guidelines [18]. Online single-breath analysis (ECOMEDICS, Duernten, Switzerland) was used with the requirement of an expiratory flow rate of 50 mL/s for a minimum of 2-s during at least a 4-s expiration time. A flow limiter maintaining constant minimum exhalation pressure of 5 cm H_2_O prevented nasal nitric oxide (NO) measurement. Measurements were repeated until criteria were met (2 results within 5 % or 3 within 10 %) and the mean was recorded.

The open-labelled food challenge to peanut was conducted according to Australasian Society of Clinical Immunology and Allergy (ASCIA) food challenge protocol [19]. A medical officer, blinded to the results from the pre-challenge clinic, supervised all challenges. Challenges were declared successful if there was no reaction during the food challenge and throughout the following week with regular ingestion of peanut. Challenges were declared unsuccessful if they had (1) anaphylaxis [which was defined according to ASCIA guidelines [20] or (2) clinical allergy, not anaphylaxis (CANA) when they demonstrated an IgE mediated reaction consistent with published pre-defined objective criteria [21].

### Follow-up cohort assessment

The follow-up study assessment was conducted over a six-month period and ranged from fifteen to thirty-two months after the individual participant’s original assessment. The assessment was conducted in the paediatric outpatients department of a tertiary children’s hospital and involved two stages—(1) undertaking the previously validated questionnaire [13, 14] (assessing the current degree of atopic disease and their family history of atopy) and —(2) SPT to whole peanut extract and purified Ara h2 protein, measuring FeNO, and blood collection to measure sIgE to peanut and Ara h2 (as described above).

### Statistical methods

STATA 13.1 and GraphPad Prism 6.0 were used for statistical evaluation and graphical presentation. Participant clinical features are presented as medians with minimum and maximum values for continuous variables (due to non-normal distribution), and frequency and percentages for categorical variables. Differences in participant clinical features between groups (defined by the results of their open food challenge in the original study) were tested using Mann–Whitney two-tailed test for continuous variables and Fisher’s exact test for categorical variables.

Receiver Operator Characteristic (ROC) curves were produced in STATA 13.1 and used to assess the ability of each measure in predicting an allergic reaction to peanuts. The area under the curve (AUC) is a summary measure of the sensitivity and specificity of the measure for all possible cut points.

Reproducibility was assessed using repeatability coefficient (C_R_) (calculated using Bland–Altman test in GraphPad Prism) and the Intra-class Correlation Coefficient (ICC) was calculated using a one-way random effects model in STATA 13.1.

## Results

### Participant clinical features

The median time between the original and follow-up measurements was 2.2 years. The clinical features are outlined in Table 1. There were no significant differences when comparing the follow-up cohort with those who did not return for follow-up. (Additional file 1: Table S1).

**Table 1:** Participant clinical features of the follow-up cohort

		Follow-up (n = 26)
Age (years)	Median (min, max)	9.4 (4.1, 17.8)
Sex (%)	Males	18 (69)
Parental smokers (%)	Total	3 (12)
Previous adrenaline required (%)	Total	6 (23)
Other food allergy (%)	Total	11 (42)
Allergic rhinitis (%)	Total	17 (65)
AR severity for those with AR—max = 4^a^	Median (min, max)	4 (1, 4)
Eczema ever (%)	Total	22 (85)
Eczema active treatment (%)	Total	12 (46)
SCORAD for those with visible eczema	Median (min, max)	10.9 (3.0, 28.9)
Asthma ever (%)	Total	17 (65)
Current preventer (%)	Total	12 (46)
Current reliever (%)	Total	15 (58)
Anaphylaxis in challenge (%)	Total	5 (19)
CANA in challenge (%)	Total	9 (35)
No allergy in challenge (%)	Total	12 (46)
Ara h2 SPT (mm)	Median (min, max)	3.8 (0.0, 9.0)
Peanut SPT (mm)	Median (min, max)	6.3 (0.0, 13.0)
Ara h2 sIgE (kU/L)	Median (min, max)	0.66 (0.00, 22.10)
Peanut sIgE (kU/L)	Median (min, max)	0.99 (0.01, 35.60)
FeNO (p.p.b)^b^	Median (min, max)	24.3 (2.7, 119.2)

One patient had an equivocal result at challenge and was excluded from the analysis

*AR* allergic rhinitis; *SCORAD* SCORing Atopic Dermatitis; *CANA* clinical allergy not anaphylaxis; *SPT* skin prick test; *sIgE* serum-specific IgE; *FeNO* fraction of exhaled nitric oxide

^a^ For determination of rhinitis severity, see ‘‘Methods’’ section

^b^ Only 22 individuals in the follow-up cohort were able to perform FeNO

### Clinical features of follow up cohort

In the follow-up cohort there were statistically significant differences between the successful [no clinical allergy (CA) at food challenge] and unsuccessful (CA at food challenge) groups in regards to age, male sex ratio, previous adrenaline usage, and current use of a preventer for asthma (Table 2). The patients without CA were younger (*P* value 0.015, Table 2). There were more males in the group of children without CA (*P* value 0.036, Table 2). Previous adrenaline usage in the CA group was higher (*P* value 0.017, Table 2). There were more participants currently using a preventer for asthma in the group of children without CA (*P* value 0.045, Table 2). As expected, children with CA did not have exposure to peanuts subsequent to the challenge test (<0.0001, Table 2). Interestingly, two children who were described as tolerant (no CA at food challenge) in the original study have subsequently developed symptoms of food allergy after eating peanut subsequent to the original study and as such now avoid eating peanut. All other clinical features across the two groups did not reach statistical significance for difference.

**Table 2:** Follow-up cohort – divided by clinical allergy or not at food challenge in original study

		No CA n = 12	CA n = 14	*P* value
Age (years)	Median (min, max)	6.8 (4.1, 15.9)	13.6 (4.5, 17.8)	*0.015*
Sex (%)	Males	11 (92)	7 (50)	*0.036*
Parental smokers (%)	Total	1 (8)	2 (14)	1.000
Previous adrenaline required (%)	Total	0 (0)	6 (43)	*0.017*
Other food allergy (%)	Total	6 (50)	5 (36)	0.692
AR (%)	Total	9 (75)	8 (57)	0.429
AR severity for those with AR—max = 4^a^	Median (min, max)	4 (1, 4)	3 (1, 4)	0.698
Eczema ever (%)	Total	12 (100)	10 (71)	0.478
Eczema active treatment (%)	Total	7 (58)	5 (36)	0.431
SCORAD for those with visible eczema	Median (min, max)	19.2 (3.0, 28.9)	7.4 (3.4, 24.4)	0.460
Asthma ever (%)	Total	9 (75)	8 (57)	0.429
Current preventer (%)	Total	8 (67)	3 (21)	*0.045*
Current reliever (%)	Total	8 (67)	7 (50)	0.453
Further exposure to peanut since challenge (%)	Total	12 (100 %)	0 (0 %)	*<0.0001*
Still eating peanuts at time of follow-up (%)^b^	Total	10 (83 %)	0 (0 %)	*<0.0001*
Ara h2 SPT (mm) (min, max)	Median (min, max)	2.3 (0.0, 5.0)	6.5 (2.0, 9.0)	*0.0001*
Peanut SPT (mm) (min, max)	Median (min, max)	4.0 (0.0, 8.5)	8.0 (5.0, 13.0)	*0.0004*
Ara h2 sIgE (kU/L)	Median (min, max)	0.08 (0.00, 4.79)	2.21 (0.41, 22.1)	*<0.0001*
Peanut sIgE (kU/L)	Median (min, max)	0.31 (0.01, 35.60)	2.84 (0.32, 23.3)	*0.0073*
FeNO (p.p.b)^c^	Median (min, max)	9.6 (2.7, 40.0)	42.1 (15.2, 119.2)	*0.0018*

One patient had equivocal result at food challenge and was therefore excluded from the analysis

Italics indicate statistical significance *P* < 0.05

*AR* allergic rhinitis; *No CA* no clinical allergy; *CA* clinical allergy; *SCORAD* SCORing Atopic Dermatitis; *CANA* clinical allergy not anaphylaxis; *SPT* skin prick test; *sIgE* serum-specific IgE; *FeNO* fraction of exhaled nitric oxide

^a^ For determination of rhinitis severity, see ‘‘Methods’’ section

^b^ Following the successful challenge, two children subsequently developed symptoms at home after eating peanut and now avoid eating peanut

^c^ Only 9 individuals in the No CA group were able to perform FeNO, while 12 individuals in the CA group were able to perform FeNO

### Data availability

Data for the peanut SPT were available for all twenty-seven individuals at the two time points. Only twelve individuals from the original group had data available for Ara h2 SPT, while twenty-seven individuals from the follow-up group had data available for Ara h2 SPT. Data for FeNO were available for twenty individuals at the two time points. Seven children had data missing from one or both time points due to being unable to perform single breath measurement of FeNO. Data were also available for peanut sIgE and Ara h2 sIgE for all twenty-seven individuals at the two time points. Due to an equivocal result in their challenge in the original study, one individual was excluded from the analysis of the follow-up cohort.

### Accuracy of Ara h2 SPT at predicting clinical outcome

There was a statistically significant difference between groups for Ara h2 SPT wheal size (*P* value 0.0001, Table 2). This compared with peanut SPT (*P* value 0.0004, Table 2), Ara h2 sIgE (*P* value <0.0001, Table 2), peanut sIgE (*P* value 0.0073, Table 2) and FeNO (*P* value 0.0018, Table 2).

### Clinical features of follow-up cohort stratified for severity of clinical allergy

In the follow-up cohort, when the CA group was divided into subgroups of children with anaphylaxis or clinical allergy not anaphylaxis (CANA) based upon the result of their food challenge, there were significant differences between the groups in regards to age and previous adrenaline usage (Table 3). Median age was 16.0 years in the anaphylaxis group compared to 10.9 years in the CANA group (*P* value 0.029, Table 3). Previous adrenaline usage in the anaphylaxis group was higher than the CANA group (*P* value 0.003, Table 3). All other clinical features across the two groups did not reach statistical significance for difference.

**Table 3:** Follow-up cohort – divided by severity of clinical allergy at food challenge in original study

		CANA (n = 9)	Anaphylaxis n = 5	*P* value
Age (years)	Median (min, max)	10.9 (4.5, 17.8)	16.0 (14.0, 17.4)	*0.029*
Sex (%)	Males	3 (33)	4 (80)	0.266
Parental smokers (%)	Total	2 (22)	0 (0)	0.506
Previous adrenaline required (%)	Total	1 (11)	5 (100)	*0.003*
Other food allergy (%)	Total	4 (44)	1 (20)	0.580
AR (%)	Total	5 (56)	3 (30)	1.000
AR severity for those with AR—max = 4^a^	Median (min, max)	4 (2, 4)	3 (1, 4)	0.750
Eczema ever (%)	Total	7 (78)	3 (60)	0.580
Eczema active treatment (%)	Total	4 (44)	1 (20)	0.580
SCORAD for those with visible eczema	Median (min, max)	7.4 (3.4, 24.4)	0.0 (0.0, 0.0)	N/A
Asthma ever (%)	Total	5 (56)	3 (60)	1.000
Current preventer (%)	Total	2 (22)	1 (20)	1.000
Current reliever (%)	Total	5 (56)	2 (40)	1.000
Ara h2 SPT (mm) (min, max)	Median (min, max)	7.0 (2.0, 8.0)	5.0 (3.5, 9.0)	0.541
Peanut SPT (mm) (min, max)	Median (min, max)	8.0 (5.5, 12.5)	6.0 (5.0, 13.0)	0.968
Ara h2 sIgE (kU/L)	Median (min, max)	1.15 (0.41, 14.50)	5.02 (0.80, 22.10)	0.227
Peanut sIgE (kU/L)	Median (min, max)	1.49 (0.32, 23.30)	3.88 (1.01, 21.80)	0.240
FeNO (p.p.b)^b^	Median (min, max)	28.3 (15.2, 119.2)	55.1 (15.4, 79.5)	0.631

Italics indicate statistical significance *P* < 0.05

*AR* allergic rhinitis; *SCORAD* SCORing Atopic Dermatitis; *CANA* clinical allergy not anaphylaxis; *SPT* skin prick test; *sIgE* serum-specific IgE; *FeNO* fraction of exhaled nitric oxide

^a^ For determination of rhinitis severity, see ‘‘Methods’’ section

^b^ Only 7 individuals in the CANA group were able to perform FeNO, while all 5 individuals in the anaphylaxis group were able to perform FeNO

### Accuracy of Ara h2 SPT at predicting severity of reaction at challenge

When stratifying the clinical allergy group by severity of reaction at challenge (CANA or anaphylaxis), Ara h2 SPT did not show ability to differentiate between the groups (*P* value 0.541, Table 3). This compared with peanut SPT, Ara h2 sIgE, peanut sIgE and FeNO (*P* values between 0.227 to 0.968).

### Accuracy of Ara h2 SPT at predicting clinical outcome—Allergy

**Fig. 1:**
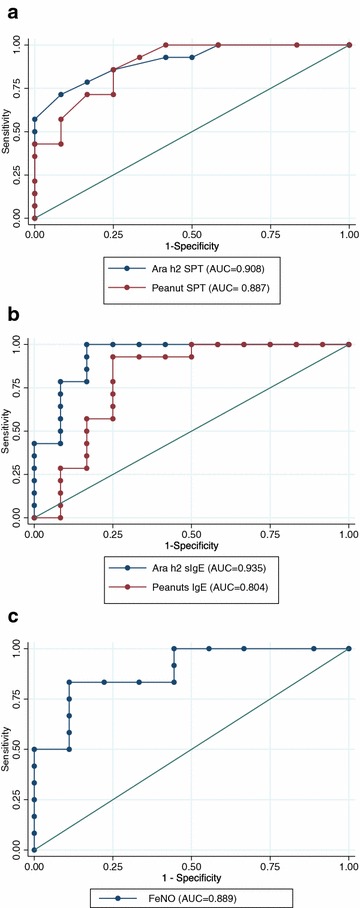
ROC curves for predicting allergy. **a** Ara h2 SPT and Peanut SPT for predicting allergy, **b** Ara h2 sIgE and Peanut sIgE for predicting allergy, **c** FeNO predicting allergy

The AUC for Ara h2 SPT predicting allergy was 0.908, which compared with the AUC for peanut SPT, Ara h2 sIgE, peanut sIgE, and FeNO for predicting allergy (0.887, 0.935, 0.804 and 0.889 respectively, Fig. 1).

### Accuracy of Ara h2 SPT at predicting clinical outcome—Anaphylaxis

**Fig. 2:**
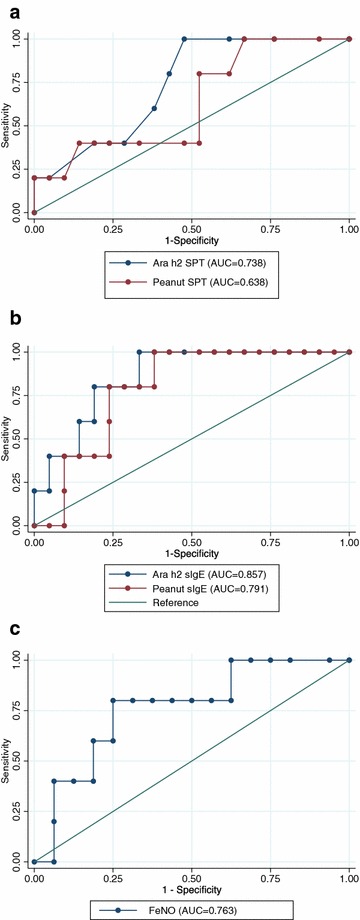
ROC curves for predicting anaphylaxis. **a** Ara h2 SPT and Peanut SPT for predicting anaphylaxis, **b** Ara h2 sIgE and Peanut sIgE for predicting anaphylaxis, **c** FeNO predicting anaphylaxis

The AUC for Ara h2 SPT predicting anaphylaxis was 0.738. This compared with the AUC for peanut SPT, Ara h2 sIgE, peanut sIgE and FeNO for predicting anaphylaxis (0.638, 0.857, 0.791 and 0.763 respectively. Figure 2).

### Reproducibility

Reproducibility for Ara h2 SPT was limited [ICC = 0.44 and C_R_ value −2.0 (Table 4)]. Peanut SPT reproducibility was also limited [ICC = 0.51 and C_R_ value 0.85 (Table 4)]. Reproducibility was higher for FeNO [ICC = 0.73 and C_R_ value −8.2 (Table 4)]. Finally, reproducibility was highest for Ara h2 sIgE [ICC = 0.85 and C_R_ value 0.03 (Table 4)] and Peanut sIgE [ICC = 0.81 and C_R_ value −0.10 (Table 4)].

**Table 4:** Reproducibility of tests

Variables	Ara h2 SPT (n = 12)	Peanut SPT (n = 26)	FeNO (n = 19)	Ara h2 sIgE (n = 26)	Peanut sIgE (n = 26)
Subgroup assessment (median & min, max)	6.5 (0.0, 10.0)	6.3 (0.0, 9.0)	32.5 (4.6, 170.5)	0.35 (0.00, 17.90)	0.90 (0.01, 31.60)
Repeat assessment (median & min, max)	3.5 (0.0, 7.5)	6.3 (0.0, 13.0)	26.6 (5.2, 119.2)	0.66 (0.00, 22.10)	0.99 (0.01, 35.60)
*P* value	0.039	0.170	0.332	0.974	0.259
*C*_*R*_ value (95 % limits of agreement)	−2.0 (−8.0, 3.9)	0.85 (−4.9, 6.6)	−8.2 (−59, 42)	0.03 (−6.1, 6.2)	−0.10 (−10.9, 10.7)
ICC value (95 % CI)	0.44 (0.00, 0.90)	0.51 (0.23, 0.80)	0.73 (0.51, 0.94)	0.85 (0.75, 0.96)	0.81 (0.68, 0.95)

## Discussion

This study demonstrates that Ara h2 SPT had similar accuracy to Peanut SPT, FeNO, Peanut sIgE, and Ara h2 sIgE at predicting allergic reaction at food challenge. While the accuracy of Ara h2 SPT was also similar with Peanut SPT, FeNO, Peanut sIgE and Ara h2 sIgE at predicting severity of reaction at food challenge, the AUC for that question is too low to be clinically useful. Larger population numbers would need to be studied to determine appropriate thresholds of Ara h2 SPT for diagnosis of peanut allergy or anaphylaxis.

To further clarify the utility of FeNO at predicting clinical allergy, we re-calculated the area under the ROC curve after excluding patients with a history of asthma but it remained unchanged (0.90). We acknowledge that this analysis is based on small numbers and future studies are required to confirm our observations.

Overall the accuracy of all tests for predicting allergic reaction at food challenge was higher than that previously reported [5, 7]. It is possible that a selection bias contributed to this result because only 48 % of the original cohort participated in the follow-up visit despite our very best recruitment efforts. Everyone who participated in the follow up study lived within 50 km of the research centre, while the original study included participants from up to 250 km away from the research centre providing some clue as why recruitment may have been less successful. It could be hypothesised that those with persisting allergy were more likely to return, as they are likely to have ongoing contact with the clinical team (some of whom were involved in the research) or having persisting allergy may make them more likely to contribute with the desire of improving diagnosis and management of peanut allergy. Thus in clinical practice all tests can be expected to have a lower predictive value than found in this study due to a regression to the mean phenomenon [22].

This study has demonstrated relatively poor reproducibility for both Ara h2 SPT and peanut SPT. This possibly relates to different operators performing the SPT in each cohort as would commonly happen in clinical practice. The ASCIA SPT manual highlights the likelihood of operator dependant technique significantly affecting SPT results [17]. Another possible cause for this poor reproducibility may be due to changes in SPT size related to further exposure to peanut, for instance by inclusion into regular diet or by accidental exposures. However, we did not observe such great variability in sIgE. Recent research on the natural history of SPT would suggest that those with persisting clinical allergy would have increasing SPT wheal size, while those with resolved clinical allergy would have decreased [23].

Despite the poor reproducibility of both Ara h2 SPT and peanut SPT, this study has demonstrated high levels of reproducibility for FeNO, and for Ara h2 sIgE and peanut sIgE serum levels. The excellent reproducibility of the sIgE results is likely contributed to by the lack of potential variation in operator technique affecting the serum sIgE result. A significant limitation remains the small population and that it may not be representative of the true value in the total population.

Two results of interest relate to the participants who appear to have lost tolerance to peanut after a successful challenge in the original study. While one participant’s peanut and Ara h2 SPT and sIgE results have decreased compared to the original challenge, the other participant’s peanut and Ara h2 SPT and sIgE results have increased. These seemingly contradictory results do not provide insight into why these two participants have developed symptoms of allergy after successful challenge (and therefore presumed tolerance) in the original study. While it is known that peanut sIgE tends to increase in children with persisting peanut allergy with repeated exposure to peanut [24], there is no published data of the natural history of peanut sIgE or Ara h2 sIgE results in children previously sensitised to peanut whose clinical allergy has resolved.

An obvious weakness of this study is the time that has passed since the food challenge with no repeat conducted on the second visit. Therefore we cannot be certain if each individual is still allergic based upon that original challenge. Those with unsuccessful challenges reported no further accidental exposures since the challenge, and all but two children who had successful challenges were continuing to eat peanut in their diet regularly. This unfortunately does not help clarify their current allergy status. However, with no further clinical indication for food challenge arising in that period, we did not believe that a repeat food challenge at that time would have been ethically sound in this cohort of children.

Another limitation is the use of open challenges in the original study. While double-blind placebo-controlled food challenges are the gold standard for diagnosing food allergy [6], in clinical practice, open-labelled food challenges are routinely used [2, 7]. In the original study [7], to help minimise the chance of false positive results, outcomes were designated based upon pre-defined objective criteria [5, 25, 26], as participants were recruited sequentially from a list of children referred clinically for open-labelled food challenge at a tertiary referral paediatric allergy centre to alleviate the lack of a placebo-controlled challenge [7].

## Conclusion

In summary, Ara h2 SPT was no worse in accuracy when compared with current testing for the evaluation of peanut allergy in this population of children. SPT with purified Ara h2 protein and peanut protein in this study demonstrate poor reproducibility and further studies could help determine inter-and intra-operator variability. FeNO demonstrated high accuracy and good reproducibility. Finally, peanut and Ara h2 sIgE collected in this study demonstrate high accuracy and excellent reproducibility over time, reaffirming the utility of these markers in assessing peanut allergy.

## Additional file

10.1186/s13223-016-0143-z Comparison of original cohort—divided by whether returned for follow-up.

## Abbreviations

ARIA: allergic rhinitis and its impact on asthma
ASCIA: Australasian Society of Clinical Immunology and Allergy
ATS/ERS: American Thoracic Society and European Respiratory Society
AUC: area under the curve
CA: clinical allergy
CANA: clinical allergy, not anaphylaxis
C_R_: coefficient of repeatability
FeNO: fraction of exhaled nitric oxide
ICC: intra-class correlation coefficient
NO: nitric oxide
ROC: receiver operator characteristic
SCORAD: SCORing Atopic Dermatitis
sIgE: specific immunoglobulin E
SPT: skin prick test
