# Supplementary material for: Reproducibility of serum IgE, Ara h2 skin prick testing and fraction of exhaled nitric oxide for predicting clinical peanut allergy in children
Source: Allergy Asthma Clin Immunol. 2016 Aug 5;12:35. doi: 10.1186/s13223-016-0143-z (PMC4975907; doi:10.1186/s13223-016-0143-z)
Supplement: Supplementary file 1 — 10.1186/s13223-016-0143-z Comparison of original cohort—divided by whether returned for follow-up. [file 13223_2016_143_MOESM1_ESM.pdf]

Supplement table 1

Comparison of original cohort – divided by whether returned for follow-up

	Values	Not followed up (n=29)	Follow-up (n=27)	*P*- value
**Age (years)**	**Median (Min, Max)**	7.8 (2.2, 15.2)	8.8 (2.1, 16.1)	0.748
**Sex**	**Males**	13 (45%)	19 (70%)	0.064
**Parental Smokers (%)**	**Total**	6 (21)	3 (11)	0.472
**Previous Adrenaline required (%)**	**Total**	2 (7)	3 (11)	0.664
**Other food allergy (%)**	**Total**	14 (48)	15 (56)	0.605
**Allergic Rhinitis (%)**	**Total**	13 (45)	13 (48)	1.000
**AR severity for those with AR - max =4**[1]	**Median (Min, Max)**	2 (1, 4)	2 (1, 4)	0.429
**Eczema ever (%)**	**Total**	24 (83)	20 (74)	0.523
**Eczema Active treatment (%)**	**Total**	9 (31)	11 (41)	0.579
**SCORAD for those with visible eczema**	**Median (Min, Max)**	26.0 (11.5, 46.0)	19.0 (15.5, 42.0)	0.977
**Asthma ever (%)**	**Total**	23 (79)	16 (56)	0.148
**Current preventer (%)**	**Total**	12 (41)	11 (41)	1.000
**Current Reliever (%)**	**Total**	21 (72)	15 (56)	0.266
**Anaphylaxis in challenge (%)**	**Total**	5 (17)	5 (19)	1.000
**CANA in challenge (%)**	**Total**	4 (15)	9 (33)	0.117
**No allergy or equivocal result in challenge (%)**	**Total**	20 (69)	13 (48)	0.174
**Ara h2 SPT (mm)**[2]	**Median (Min, Max)**	4.5 (0.0, 9.0) (n=13)	6.5 (0.0, 10.0) (n=12)	0.490
**Peanut SPT (mm)**	**Median (Min, Max)**	6.0 (0.0, 11.0)	6.5 (0.0, 9.0)	0.772
**Ara h2 sIgE (kU/L)**	**Median (Min, Max)**	0.35 (0.00, 100.00)	0.35 (0.00, 98.10)	0.798
**Peanut sIgE (kU/L)**	**Median (Min, Max)**	0.77 (0.02, 100.00)	0.93 (0.01, 100.00)	0.646
**FeNO (p.p.b)**[3]	**Median (Min, Max)**	27.4 (4.5, 67.4) (n=26)	32.0 (3.9, 170.5) (n=21)	0.417

[1] For determination of rhinitis severity, see Methods.

[2] Only 12 individuals in the follow-up cohort had Ara h2 SPT measured, while only 13 individuals in the not followed-up cohort had Ara h2 SPT measured.

[3] Only 21 individuals in the follow-up cohort were able to perform FeNO, while only 26 individuals in the not followed-up cohort were able to perform FeNO.

AR, allergic rhinitis; SCORAD, SCORing Atopic Dermatitis; CANA, Clinical Allergy Not Anaphylaxis; SPT, Skin Prick Test; sIgE, serum-specific IgE; FeNO, Fraction of exhaled Nitric Oxide.
